# 5-Hydroxytryptophan acts as a gap junction inhibitor to limit the spread of chemotherapy-induced cardiomyocyte injury and mitochondrial dysfunction

**DOI:** 10.18632/aging.205641

**Published:** 2024-03-10

**Authors:** Wenshe Sun, Qi Lu, Yukun Zhang, Dongming Xing

**Affiliations:** 1 Qingdao Cancer Institute, Qingdao University, Qingdao 266071, China; 2 The Affiliated Hospital of Qingdao University and Qingdao Cancer Institute, Qingdao 266071, China

**Keywords:** chemotherapy, cardiotoxicity, 5-hydroxytryptophan, gap junction, collagen

## Abstract

Anthracycline chemotherapeutics like doxorubicin (DOX) are widely used against various cancers but are accompanied by severe cardiotoxic effects that can lead to heart failure. Through whole transcriptome sequencing and pathological tissue analysis in a murine model, our study has revealed that DOX impairs collagen expression in the early phase, causing extracellular matrix anomalies that weaken the mechanical integrity of the heart. This results in ventricular wall thinning and dilation, exacerbating cardiac dysfunction. In this work, we have identified 5-hydroxytryptophan (5-HTP) as a potent inhibitor of gap junction communication. This inhibition is key to limiting the spread of DOX-induced cardiotoxicity. Treatment with 5-HTP effectively countered the adverse effects of DOX on the heart, preserving ventricular structure and ejection fraction. Moreover, 5-HTP enhanced mitochondrial respiratory function, as shown by the O2k mitochondrial function assay, by improving mitochondrial complex activity and ATP production. Importantly, the cardioprotective benefits of 5-HTP did not interfere with DOX’s ability to combat cancer. These findings shed light on the cardiotoxic mechanisms of DOX and suggest that 5-HTP could be a viable strategy to prevent heart damage during chemotherapy, offering a foundation for future clinical development. This research opens the door for 5-HTP to be considered a dual-purpose agent that can protect the heart without compromising the oncological efficacy of anthracycline chemotherapy.

## INTRODUCTION

Anthracycline-based chemotherapy, exemplified by doxorubicin, remains a linchpin in the therapeutic arsenal against a spectrum of malignancies, including breast cancer, due to its superior efficacy [1–4]. However, the emergence of chemotherapy-induced cardiomyopathy is a critical concern, accounting for significant mortality rates—up to 30%—among cancer patients [5, 6]. The intricate mechanisms underpinning DOX-induced cardiotoxicity (DIC) are not fully delineated, prompting a call for more comprehensive research to decode these pathways. Dexrazoxane (Dextra), a stereoisomer of razoxane and chemically designated as (S)-(+)-4,4′-(1-methyl-1,2-ethanediyl) bis (2,6-piperazinedione), represents the sole FDA-endorsed agent for mitigating anthracycline-induced cardiac damage. Marketed as Zinecard by Pharmacia and Upjohn and later as Aoxinoxian for domestic use, Dextra operates as an iron-chelating entity, thwarting free radical genesis and catalyzing the swift breakdown of topoisomerase IIβ. This intervention has been clinically shown to diminish both the frequency and severity of DOX-related cardiotoxicity, specifically recommended for women with metastatic breast cancer who have surpassed a cumulative DOX dosage of 300 mg/m^2^ and are deemed suitable for continued chemotherapy. Despite its benefits, the pronounced side effects of Dextra necessitate the pursuit of more benign and efficacious cardioprotective modalities [7–9].

Gap junctions, comprising conglomerates of intercellular channels, are pivotal for the synchronized cardiac function, enabling electrical and metabolic crosstalk between myocytes [10]. Beyond their role in excitable cells, such as neurons and cardiomyocytes, gap junctions are instrumental in non-excitable tissues, orchestrating cellular growth, differentiation, and the maintenance of homeostasis [11]. Disruptions in gap junction integrity are implicated in a plethora of pathologies, ranging from cardiac failure to oncogenesis. Prevailing evidence indicates that gap junction-mediated signaling is instrumental in propagating ischemic-reperfusion injury across myocardial cells, precipitating pronounced contractility issues and cellular demise [10, 12]. Preemptive attenuation of gap junction connectivity has been shown to substantially mitigate the extent of infarction [13, 14]. Nevertheless, the potential of gap junction modulation as a protective strategy against chemotherapeutic-induced cardiac injury remains an uncharted scientific endeavor.

5-Hydroxytryptophan (5-HTP), isolated from the seeds of the West African plant Griffonia simplicifolia, serves as a biochemical precursor to serotonin (5-hydroxytryptamine, 5-HT) and plays a critical role in the regulation of cardiac morphogenesis [15]. Emerging research suggests that 5-HTP may surpass melatonin in its antioxidative capacity, conferring enhancements in both myocardial contractility and chronotropic responsiveness [16–18]. Despite these findings, the cardioprotective potential of 5-HTP against anthracycline-induced cardiotoxicity remains an open question.

The present investigation provides compelling evidence that 5-HTP can effectively modulate cardiomyocyte gap junction dynamics, thereby mitigating doxorubicin (DOX)-induced mitochondrial dysfunction and subsequent myocardial injury. Utilizing both *in vivo* murine models of acute myocardial injury induced by DOX and *in vitro* H9c2 cardiomyocyte injury assays, our study delineates a novel cardioprotective mechanism of 5-HTP, positioning it as a promising therapeutic candidate for ameliorating the cardiotoxic effects of anthracycline chemotherapy.

## MATERIALS AND METHODS

### Reagents

Doxorubicin and dexrazoxane were procured from Hubei Weidely Chemical Technology Co., Ltd., while 5-hydroxytryptophan was sourced from Xi’an EnTaiYuan Biotechnology Co. The cell culture essentials, including Dulbecco’s Modified Eagle Medium (DMEM), fetal bovine serum (FBS), trypsin, penicillin/streptomycin, and the cell counting kit-8 (CCK-8) reagent, were obtained from Dalian Meilun Biotechnology Co. Histological and biochemical assay kits, such as those for hematoxylin and eosin (H&E) staining, Fura-2 AM probe, lactate dehydrogenase (LDH), malondialdehyde (MDA), DCFH-DA probe, and Masson’s trichrome staining, were supplied by Shanghai Biyuntian Biotechnology Co. and Beijing Solaibao Technology Co. Additionally, primary antibodies against interleukin-1 beta (IL-1β) and horseradish peroxidase (HRP)-conjugated secondary antibodies were purchased from Wuhan ABclonal Biotechnology Co.

### Cell culture and animals treatments

Rat H9c2 cardiomyocytes, human MCF-7 breast cancer cells, and human U2OS osteosarcoma cells were procured from the American Type Culture Collection (ATCC; Manassas, VA, USA). These cells were cultured in DMEM supplemented with 10% FBS and 1% penicillin/streptomycin, and maintained at 37°C in a humidified atmosphere containing 5% CO_2_. Upon reaching 70–80% confluence, the cells were treated with the respective drugs for an additional 24 hours. Based on prior research, a concentration of 5 μM DOX was identified as optimal for *in vitro* induction of myocardial injury, while 20 μM dexrazoxane exhibited significant cardioprotective effects against DOX-induced damage. Cell viability was assessed using CCK8 across a range of 5-HTP concentrations (5, 10, 20, 50, 100 μM), revealing that 20 μM 5-HTP effectively counteracted the DOX-induced decline in cell viability. The experimental groups were defined as follows: Control (untreated cells), DOX (cells treated with 5 μM DOX), Dexra+DOX (cells treated with 5 μM DOX and 20 μM dexrazoxane), and 5-HTP+DOX (cells treated with 5 μM DOX and 20 μM 5-HTP).

Forty-eight female C57BL/6J mice, aged 8 weeks and weighing approximately 18 ± 2 g, were obtained from SPF (Beijing) Biotechnology Co., Ltd. These animals were housed under specific pathogen-free (SPF) conditions at the Qingdao University animal testing center, with ad libitum access to food and water. The experimental protocol commenced following a one-week acclimatization period and received formal approval from the Experimental Animal Welfare Ethics Committee of Qingdao University (Approval No. 20211202C577620211217068).

In this investigation, forty-eight female C57BL/6J mice were stratified into six cohorts, each comprising eight individuals: a control group receiving saline (Control), a group treated with doxorubicin (DOX), a group receiving both dexrazoxane and doxorubicin (Dexra+DOX), and three groups subjected to low, medium, and high doses of 5-hydroxytryptophan in conjunction with doxorubicin (L-5-HTP+DOX, M-5-HTP+DOX, H-5-HTP+DOX, respectively). The experimental timeline spanned seven days, with a single doxorubicin dose of 10 mg/kg delivered intraperitoneally on day one to induce acute myocardial injury. Dexrazoxane was administered intraperitoneally at a daily dose of 14.3 mg/kg, reaching a cumulative dose 10-fold that of doxorubicin (100 mg/kg). The administration of 5-HTP via oral gavage occurred daily at doses of 7.5 mg/kg, 15 mg/kg, and 30 mg/kg for the low, medium, and high dose groups, respectively. These dosages were selected based on their efficacious use in prior studies [19–21].

Throughout the duration of the study, the body weight of each mouse was meticulously recorded on a daily basis. At the study’s conclusion, the mice were humanely euthanized under deep anesthesia induced by ketamine (75 mg/kg) and xylazine (10 mg/kg) to ensure minimal suffering. Post-euthanasia, the hearts were excised and subjected to photographic documentation. This approach to euthanasia aligns with the highest standards of animal welfare, aiming to significantly reduce the duration of potential pain and distress experienced by the animals.

### Echocardiography

On day 8 of the experiment, mice were anesthetized with 1.5% isoflurane and left ventricular ejection fraction, left ventricular fractional shortening, left ventricular internal diastolic end-systolic, left ventricular internal diastolic end-diastolic, and heart rate (HR) were measured using an ultrasound imaging system (V6, Vinno China) to assess the cardiac function of the mice. The left ventricular end-systolic diameter (LVESd) and left ventricular end-diastolic diameter (LVEDd).

### Histological staining

Mice hearts were fixed in 4% paraformaldehyde, dehydrated, and embedded in paraffin, and 4 μm thick sections were obtained using a microtome. The sections were stained with hematoxylin and eosin, and MASSON staining according to the kit instructions to observe the morphological changes in the myocardial tissue under a light microscope (Nikon, Japan).

Sections were immunohistochemically stained using IL-1β antibody to observe changes in inflammatory factors in the heart tissue. Sections were incubated for 10 min at room temperature using 3% H_2_O_2_ solution, 5% goat serum was used for closure, and IL-1β antibody dilution (1:200) was added dropwise to the tissue sections and incubated overnight at 4°C. The following day, the sections were incubated with secondary antibody at 37°C for 15 min, rinsed in PBS, and stained with drops of DAB chromogen. Then the nuclei were stained using hematoxylin to observe the expression of inflammatory factor IL-1β in different groups under a microscope (Nikon, Japan).

### RNA sequencing and differential gene enrichment analysis

Cardiac tissues from the experimental cohorts—Control, DOX-treated, and H-5-HTP-protected mice—were rapidly excised and immediately submerged in liquid nitrogen to preserve RNA integrity. Subsequent processing adhered strictly to the manufacturer’s protocols for RNA extraction, reverse transcription, and DNA amplification. High-throughput sequencing was performed on an Illumina NovaSeq 6000 platform. Differential gene expression analysis was conducted with stringent criteria: a *p*-value threshold of ≤0.05 and an absolute log2 fold change greater than 0. To elucidate the biological pathways impacted by differential gene expression, we employed both KEGG and Reactome enrichment analyses to identify significant pathways.

### Measurement of mitochondrial respiratory function in cardiac tissue

Three mouse hearts each from the Control, DOX, and H-5-HTP protected groups were taken, and half of the dissected hearts were snap-frozen in liquid nitrogen to determine other indices. The other half was weighed and ground using a tissue grinder for high-resolution respiration measurements. Mitochondrial respiration (state 4), i.e., conventional mitochondrial respiration, was initiated by adding 5 mM pyruvate (P) and 2 mM malate (M) to the chamber of the Oxygraph-2k instrument (Oroboros, Austria) [22]; then 2.5 mM ADP and 3 mM MgCl_2_ were added to activate ATP synthase and record mitochondrial oxidative phosphorylation (OXPHOS) levels (state 3) [23]; followed by the addition of 5 nM oligomycin (Omy), which inhibits ATP synthase to induce a non-phosphorylated leaky state and estimate overall mitochondrial-associated respiration [22]. Finally, the addition of 2.5 μM antimycin A (Ama), which inhibits complex III activity and thus shuts down mitochondrial oxygen consumption, was used to assess the residual oxygen consumption by subtracting this fraction, was used to obtain the oxygen consumption due to mitochondrial respiration at different stages and thus to evaluate the mitochondrial function. The respiratory control rate (RCR) can be calculated by calculating the state 3/state 4 oxygen consumption rate ratio.

### Measurement of intracellular reactive oxygen species (ROS)

To assess the cellular oxidative stress response, H9c2 cardiomyoblasts seeded on 6-well plates were subjected to the respective group treatments. The intracellular ROS levels were quantified using the 2’,7’-dichlorodihydrofluorescein diacetate (DCFH-DA) fluorescent probe. Post-treatment, cells were rinsed with serum-free media and incubated with 20 μM DCFH-DA for 20 minutes. Fluorescence was visualized and captured using a confocal microscope (Nikon, Japan). The fluorescence intensity was quantitatively analyzed using ImageJ software to determine relative ROS levels.

### Measurement of intracellular calcium ion levels

We measured the intracellular Ca2+ concentrations utilizing the Fura-2 AM fluorescent probe. H9c2 cells, treated with various pharmacological agents, were incubated with 0.5 μM Fura-2 AM at 37°C for 60 minutes. Following incubation, cells were washed with phosphate-buffered saline (PBS) and imaged under a fluorescence microscope (Leica, Germany) to determine the intracellular Ca2+ levels.

### Spectrophotometric analysis of cellular biomarkers

The concentrations of malondialdehyde (MDA) and lactate dehydrogenase (LDH) in H9c2 cells were determined via colorimetric assays. Cells were lysed, and the MDA levels were spectrophotometrically assessed, with absorbance readings taken using a PE Victor Nivo 3S microplate reader. LDH release, an indicator of cellular damage, was measured using an assay kit (Solarbio, Beijing, China) following the manufacturer’s protocol. LDH levels were quantified from the culture medium of cells grown to ~80% confluency and subjected to drug treatment for 24 hours. Absorbance was recorded on a microplate reader.

### Overexpressing Cx43 in cardiac myocytes

To investigate the impact of Cx43 overexpression on cardiotoxicity, we designed and constructed a plasmid for overexpressing Cx43 in cardiac myocytes. This plasmid contains the full coding sequence of Cx43. Additionally, the plasmid includes an antibiotic resistance gene for selecting cells that have been successfully transfected. After transfecting the plasmid into cardiac myocytes via electroporation or lipofection, we verified Cx43 overexpression using techniques such as qPCR and Western blot. Subsequently, we divided the cells into various groups: control, doxorubicin (DOX), 5-hydroxytryptophan (5-HTP) + DOX, Cx43 overexpression + DOX, and 5-HTP + DOX + Cx43 overexpression, to conduct a series of experiments assessing cardiotoxicity.

### Cell viability assay of MCF-7 and U2OS cells

The viability of MCF-7 and U2OS cells post-chemical treatment was evaluated using the Cell Counting Kit-8 (CCK-8) assay. Cells were seeded at a density of 10,000 cells per well in a 96-well plate and allowed to adhere for 24 hours. Following initial culture, the medium was replaced with treatment-specific drug combinations. After a 24-hour treatment period, 10 μL of CCK-8 reagent was added to each well, and the cells were incubated at 37°C for 2 hours. Absorbance at 450 nm was measured using a microplate reader (PE Victor Nivo 3S, Waltham, MA, USA). Cell survival percentages were calculated by comparing the optical density (OD) values of the treated groups to those of the control group.

### Statistical analysis

Data were analyzed using SPSS 22.0 statistical software. Experimental results (*n* ≥ 5) were expressed as mean ± standard deviation (x ± SD), with one-way analysis of variance (ANOVA) used for significant differences between multiple groups and LSD for post hoc tests; independent samples *t*-test was used to compare the differences between the two groups, and *P* < 0.05 indicated that the differences were statistically significant.

## RESULTS

### DOX inhibits collagen synthesis in myocardium and causes progressive thinning of the ventricular wall

**Figure 1:**
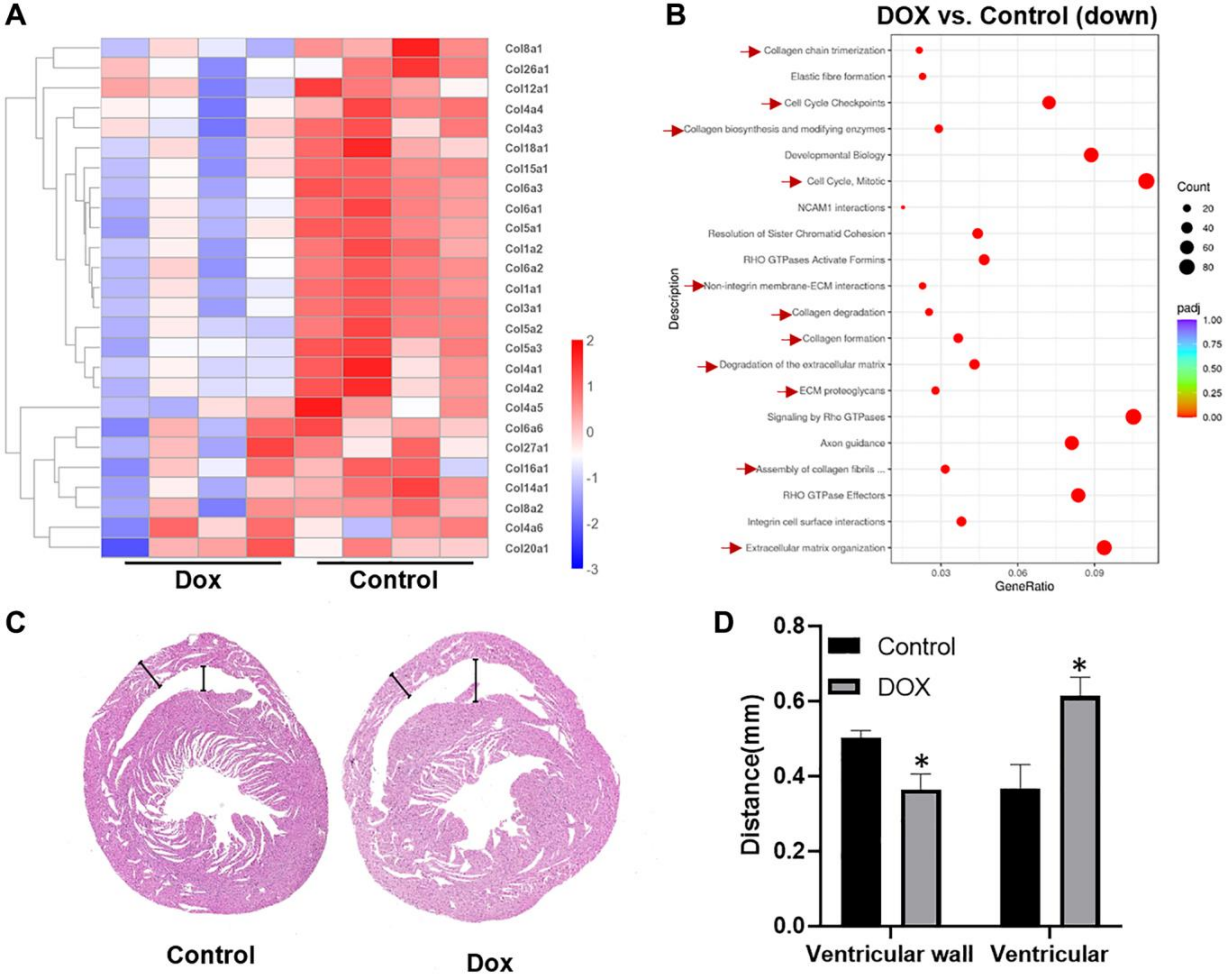
**DOX inhibits collagen synthesis in cardiomyocytes and causes progressive thinning of the ventricular wall.** (**A**) heat map of differential expressed collagen genes in the control group versus DOX group; (**B**) scatter plot of Reactome enrichment analysis of down regulated genes affected by DOX; (**C**) scan of heart tissue sections after HE staining; (**D**) statistical illustration of ventricular wall thickness and ventricular size in the control and DOX group. ^*^P < 0.05 vs. the Control group, ^#^*P* < 0.05 vs. the DOX group.

To investigate the early pathophysiological changes induced by doxorubicin (DOX) in cardiac tissue, we developed a mouse model of DOX-induced cardiomyopathy. In this model, mice were administered a single intraperitoneal injection of DOX at a dosage of 10 mg/kg. After a period of one-week, cardiac tissue samples were harvested for comprehensive transcriptomic analysis. Transcriptome sequencing results, depicted in Figure 1A, revealed a pronounced downregulation of several collagen genes in the DOX-treated cohort relative to the control group. This finding was further substantiated by Reactome pathway enrichment analysis, as illustrated in Figure 1B, which underscored not only the anticipated cell cycle arrest but also highlighted a significant impact on the biosynthesis of the extracellular matrix (ECM) and collagen within cardiac fibroblasts. Pathological examination of cardiac tissue, demonstrated in Figure 1C, 1D, provided visual confirmation of these molecular alterations. Notably, the ventricular walls of DOX-treated mice were markedly thinner, and the ventricular chambers were dilated in comparison to those of untreated controls. These morphological changes are indicative of ventricular remodeling, characterized by ECM degradation, ventricular dilation, and a reduction in ventricular wall thickness. Our observations align with the pathological sequelae of myocardial infarction reported by Takahashi et al., who documented a significant loss—approximately 50%—of myocardial collagen within the infarcted zone as early as three hours post-infarction, followed by accelerated collagen degradation during ischemic reperfusion [24]. Such parallels lead us to posit that DOX may impede the myocardial repair process in the acute phase of drug-induced cardiotoxicity (DIC) by attenuating collagen synthesis. This impairment could facilitate the propagation of cardiac injury, exacerbating the detrimental remodeling processes.

### 5-HTP mitigates doxorubicin-induced cardiac dysfunction and structural myocardial damage in mice

**Figure 2:**
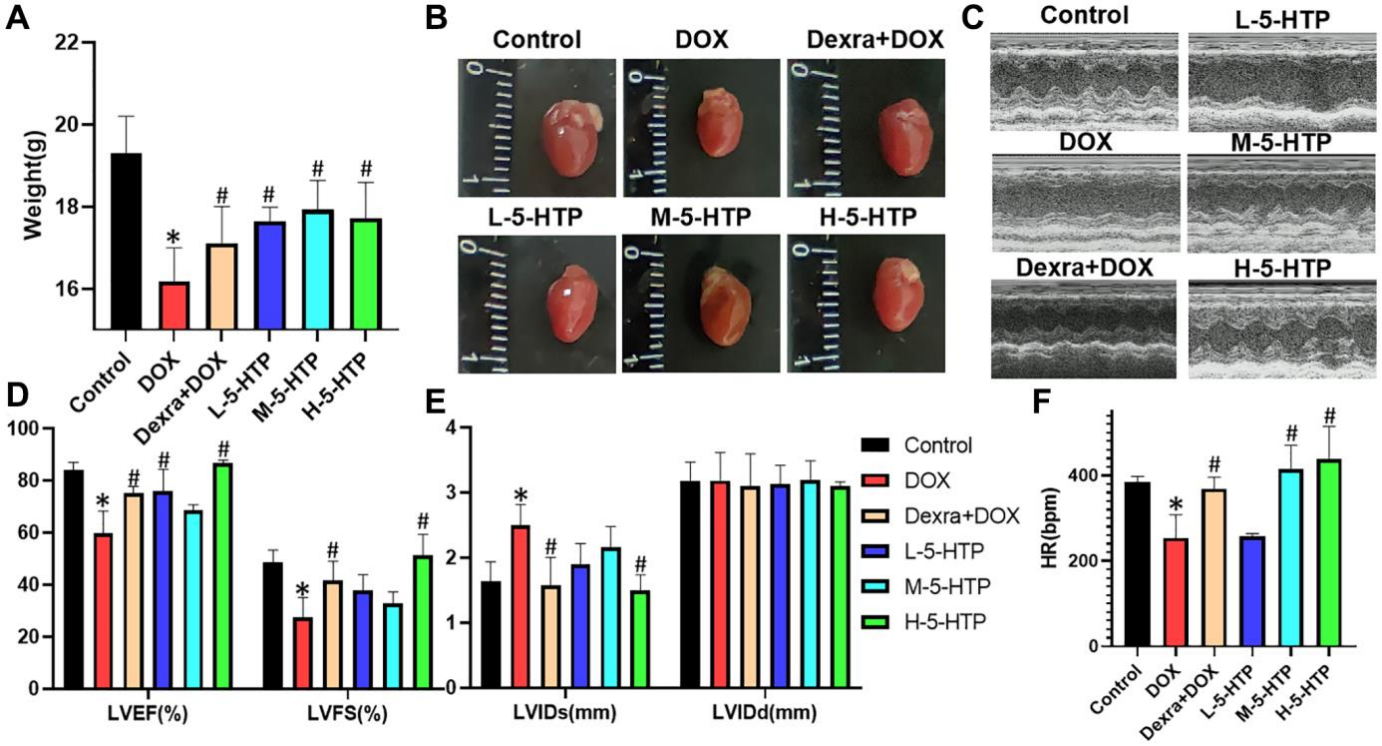
**5-HTP ameliorates DOX-induced cardiac dysfunction of myocardial tissue in mice.** (**A**) Bodyweight of mice on the last day of the experiment; (**B**) Morphology of the mice heart; (**C**) Representative echocardiographic images; (**D**) Left ventricular ejection fraction (LVEF) and left ventricular fractional shortening (LVFS); (**E**) Left ventricular internal diastolic end-systolic (LVIDs) and left ventricular internal diastolic end-diastolic (LVIDd); (**F**) Heart rate (HR); ^*^P < 0.05 vs. the Control group, ^#^*P* < 0.05 vs. the DOX group. (L-5-HTP group: L-5-HTP+DOX; M-5-HTP group: M-5-HTP+DOX; H-5-HTP group: H-5-HTP+DOX).

In our exploration of the cardioprotective properties of 5-hydroxytryptophan (5-HTP) against doxorubicin-induced cardiotoxicity (DIC), we developed an acute model of myocardial injury in mice. This was achieved by administering a single substantial dose of doxorubicin (DOX) at 10 mg/kg, concomitantly with daily intraperitoneal injections of 5-HTP. Notably, the mice subjected to DOX treatment (16.18 g ± 0.82) exhibited a significant reduction in body weight relative to the control group (19.30 g ± 0.90) (*P* < 0.05), a trend that was markedly reversed upon administration of 5-HTP (*P* < 0.05) as shown in Figure 2A. Additionally, the hearts of DOX-treated mice were visibly smaller when compared to those of the control mice (Figure 2B).

Prior to euthanasia, echocardiographic assessments were performed to evaluate cardiac function. The results indicated a drastic decline in systolic function in the DOX-treated group, evidenced by reduced left ventricular ejection fraction (LVEF), left ventricular fractional shortening (LVFS), and heart rate (HR) (*P* < 0.05), coupled with an increase in left ventricular internal dimension at systole (LVIDs). These findings suggest that DOX primarily impairs left ventricular systolic function (Figure 2C–2F). Conversely, both the clinical cardioprotective agent dexrazoxane (Dexra) and 5-HTP exhibited significant protective effects, enhancing LVEF, LVFS, and HR (*P* < 0.05). Notably, a high dose of 5-HTP (30 mg/kg) demonstrated a profound improvement in cardiac function, rivaling the efficacy of Dexra (Figure 2C–2F).

**Figure 3:**
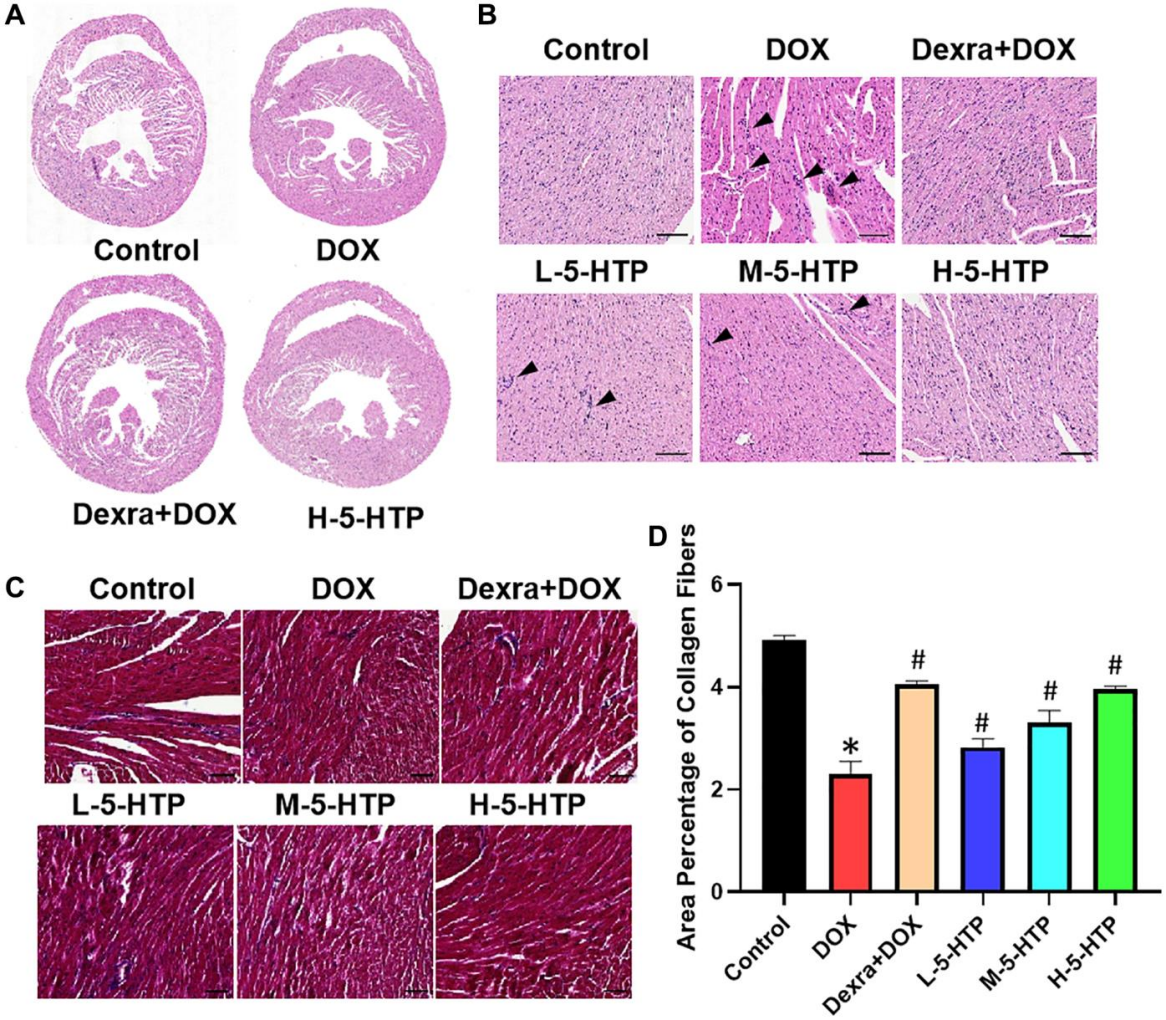
**5-HTP ameliorates DOX-induced morphological damage of myocardial tissue in mice.** (**A**) Scans of heart tissue sections; (**B**) The results from HE staining, bar = 100 μm; (**C**, **D**) The results and extent of fibrosis from MASSON staining, bar = 50 μm; ^*^P < 0.05 vs. the Control group, ^#^*P* < 0.05 vs. the DOX group. (L-5-HTP group: L-5-HTP+DOX; M-5-HTP group: M-5-HTP+DOX; H-5-HTP group: H-5-HTP+DOX).

Histopathological examination of cardiac tissue revealed that while the control group maintained normal myocardial architecture, the DOX group exhibited extensive cardiac damage. This included disruption of myofibrillar organization, structural alterations, expansion of interstitial spaces, and degeneration of myocardial fibers (Figure 3A, 3B). Remarkably, 5-HTP treatment mitigated these deleterious effects, attenuated DOX-induced ventricular dilation, preserved ventricular wall thickness, and reinstated structural integrity of the myocardium. Furthermore, Masson’s trichrome staining of myocardial tissue indicated a reduction in interstitial fibrosis following DOX exposure, with 5-HTP treatment facilitating the reestablishment of a typical cardiac extracellular matrix (ECM) structure (Figure 3C, 3D). In summary, both Dexra and 5-HTP interventions significantly ameliorated morphological damage to cardiac tissue, with 5-HTP exhibiting a superior phenotype in the restoration of cardiac tissue normalization.

### 5-HTP attenuates doxorubicin-induced cardiotoxicity by modulating gap junction communication

**Figure 4:**
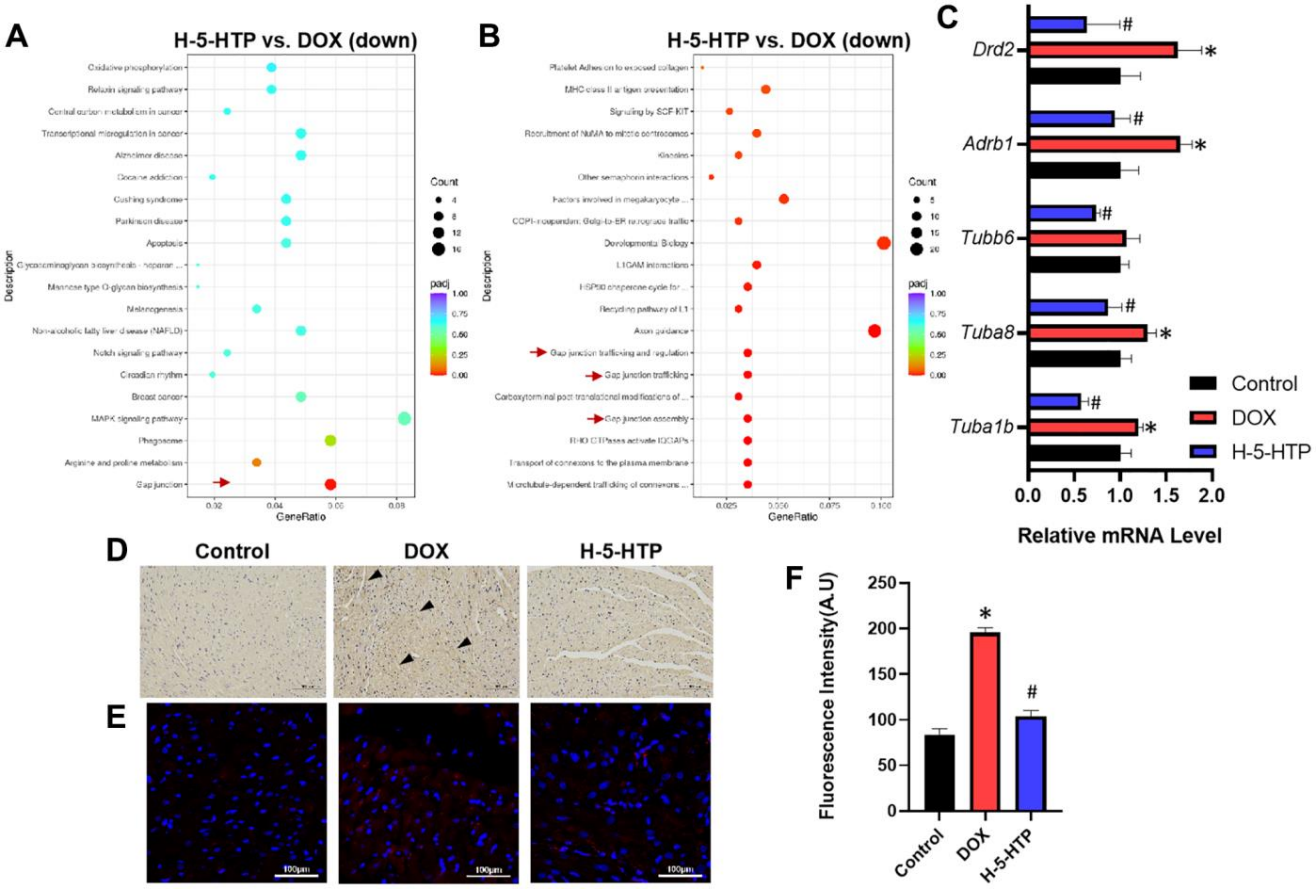
**5-HTP ameliorates doxorubicin-induced cardiotoxicity through inhibition of gap junctions.** (**A**) Scatter plot of KEGG enrichment analysis of down-regulated genes by H-5-HTP group; (**B**) Scatter plot of Reactome enrichment analysis of down-regulated genes by H-5-HTP group; (**C**) Relative levels of mRNA for gap junction-related genes; (**D**) IL-1β immunohistochemistry results, bar = 100 μm; (**E**) Cx43 immunofluorescence results, bar = 100 μm; (**F**) Fluorescence intensity of Cx43 immunofluorescence; ^*^P < 0.05 vs. the Control group, ^#^*P* < 0.05 vs. the DOX group. (L-5-HTP group: L-5-HTP+DOX; M-5-HTP group: M-5-HTP+DOX; H-5-HTP group: H-5-HTP+DOX).

To elucidate the cardioprotective mechanism of 5-hydroxytryptophan (5-HTP) against doxorubicin (DOX)-induced cardiotoxicity (DIC), we conducted a thorough analysis of the molecular pathways involved. Utilizing Kyoto Encyclopedia of Genes and Genomes (KEGG) pathway mapping (Figure 4A) and Reactome pathway enrichment analysis (Figure 4B), we compared the differentially expressed genes in the hearts of mice treated with 5-HTP to those in the DOX-only group. Our findings suggest that 5-HTP exerts its protective effects primarily by impeding gap junctional communication within the myocardium. This is noteworthy because gap junctions facilitate the paracrine signaling of ions and molecules, which, in turn, are pivotal for intercellular communication within cardiac tissue. The perturbation of these channels during ischemic events is known to exacerbate tissue damage. Corroborating the literature [13, 14], our study shows that diminishing gap junctional coupling prior to ischemic insult can significantly curtail the extent of myocardial infarction.

In line with this, we observed a marked downregulation in the expression of gap junction-related genes, including Tuba1b, Tuba8, Tubb6, Adrb1, and Drd2, following the administration of 5-HTP (Figure 4C). To further substantiate the role of 5-HTP as an inhibitor of gap junctions in preventing the propagation of DOX-induced myocardial injury, we assessed the levels of cardiac inflammation. Post-DOX treatment, there was a notable upsurge in inflammatory markers, a trend that was effectively reversed by 5-HTP, indicating a reduction in the spread of myocardial inflammation (Figure 4D). Immunofluorescence staining was carried out on heart tissues in our study, and the results also showed that the expression of non-phosphorylated Cx43 decreased after 5-HTP treatment and significantly increased after DOX treatment, indicating that DOX enhanced cardiotoxicity through GJ, while 5-HTP inhibited this occurrence (Figure 4E, 4F).

In light of the complex interplay between gap junction dynamics and calcium homeostasis in cardiomyocytes, we have refined our understanding of the mechanisms underlying doxorubicin (DOX)-induced cardiotoxicity and the protective effects of 5-hydroxytryptophan (5-HTP). The control of calcium levels is crucial in cardiomyocytes, as calcium ions play a pivotal role in regulating contractile activity. DOX exacerbates intracellular calcium levels by promoting calcium release from the sarcoplasmic reticulum, thus enhancing calcium channel opening. Gap junctions, particularly those formed by Connexin 43 (Cx43), facilitate the intercellular passage of small molecules, including Ca2+ and cyclic adenosine monophosphate (cAMP) [25], which are essential for the coordinated response of cardiomyocytes to stressors.

**Figure 5:**
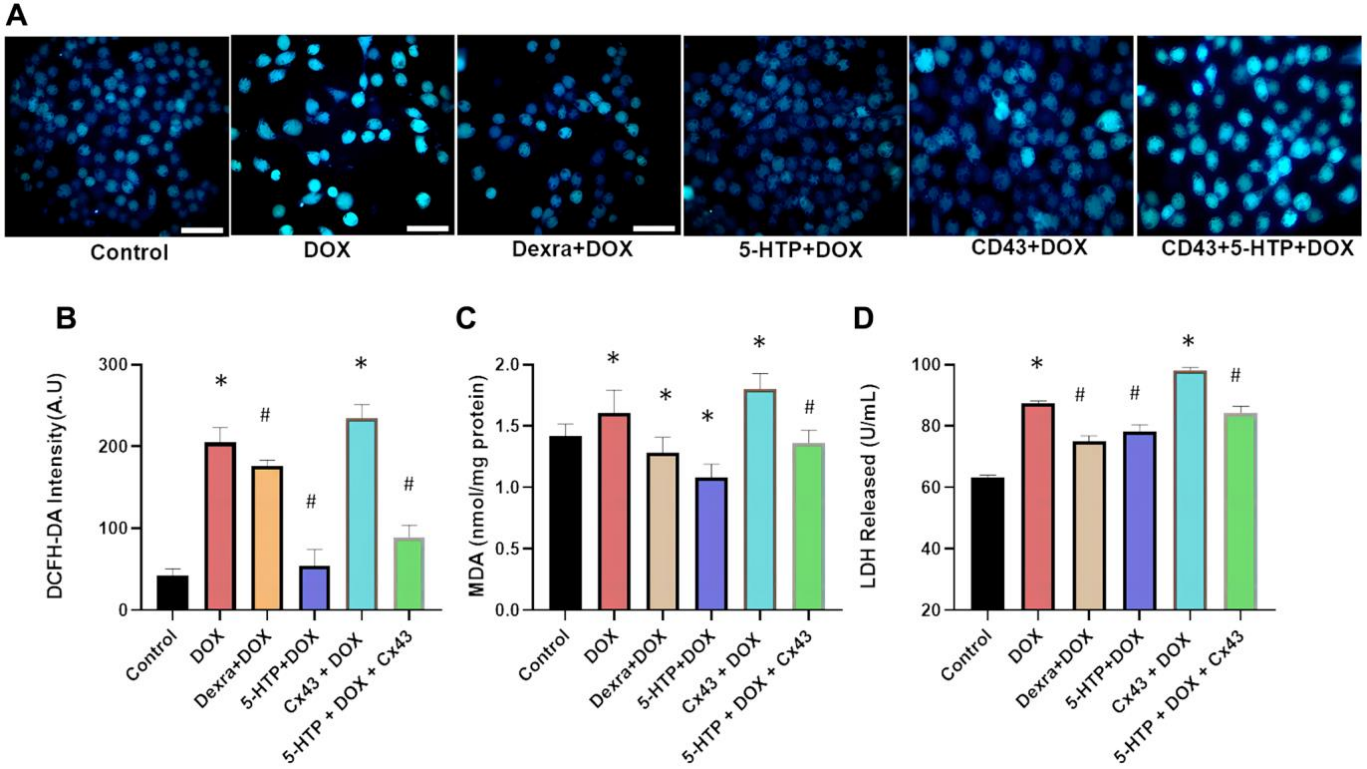
**5-HTP significantly improves doxorubicin-induced oxidative damage in H9c2 cells.** (**A**) Fluorescence microscopy image of intracellular calcium ions, bar = 100 μm; (**B**) DCFH-DA probe detects reactive oxygen species in each group of cells; (**C**) Intracellular MDA levels in different treatment groups; (**D**) LDH release in the supernatant is shown. ^*^P < 0.05 vs. the Control group, ^#^*P* < 0.05 vs. the DOX group.

Our research indicates that gap junctions may exacerbate the effects of DOX-induced calcium dysregulation, contributing to the spread of injury within the myocardium. We utilized a calcium probe to monitor cardiomyocyte calcium ion dynamics and observed a significant elevation in myocardial calcium ion signaling following DOX treatment, compared to the control. Notably, 5-HTP treatment restored calcium ion levels to normal, akin to the control condition, suggesting its potential role in mitigating DOX-induced disruptions in calcium homeostasis (Figure 5A). Further investigation into the role of gap junctions during ischemia revealed that Cx43-mediated junctions perpetuate intercellular communication, allowing the spread of contractile dysfunction and calcium overload among ischemic cardiomyocytes. DOX-induced myocardial injury similarly leads to calcium overload, with gap junctions playing a pivotal role in the diffusion of injury. Theoretically, reducing gap junction (GJ) coupling could diminish the extent of DOX-induced myocardial toxicity.

Our study’s immunofluorescence staining of heart tissues confirmed these insights, showing a decrease in non-phosphorylated Cx43 expression following 5-HTP treatment and a significant increase post-DOX treatment. This suggests that DOX amplifies cardiotoxicity through GJ enhancement, while 5-HTP inhibits this process. Additionally, markers of DOX-induced oxidative damage, such as reactive oxygen species (ROS, Figure 5B) and malondialdehyde (MDA, Figure 5C), were significantly diminished following 5-HTP treatment. The assay for lactate dehydrogenase (LDH) leakage, a marker of cardiomyocyte damage, revealed that DOX markedly increased LDH release into the medium compared with the control group (*P* < 0.05). Conversely, treatments with 5-HTP (and Dexra) effectively preserved normal cardiac structure and reduced LDH leakage after DOX-induced cellular damage (Figure 5D). In conclusion, our findings support the hypothesis that 5-HTP, by acting as a gap junction inhibitor, significantly attenuates the spread of DOX-induced damage and contributes to the maintenance of cardiac microenvironmental homeostasis.

### 5-HTP significantly improves DOX-induced mitochondrial dysfunction in mouse heart

**Figure 6:**
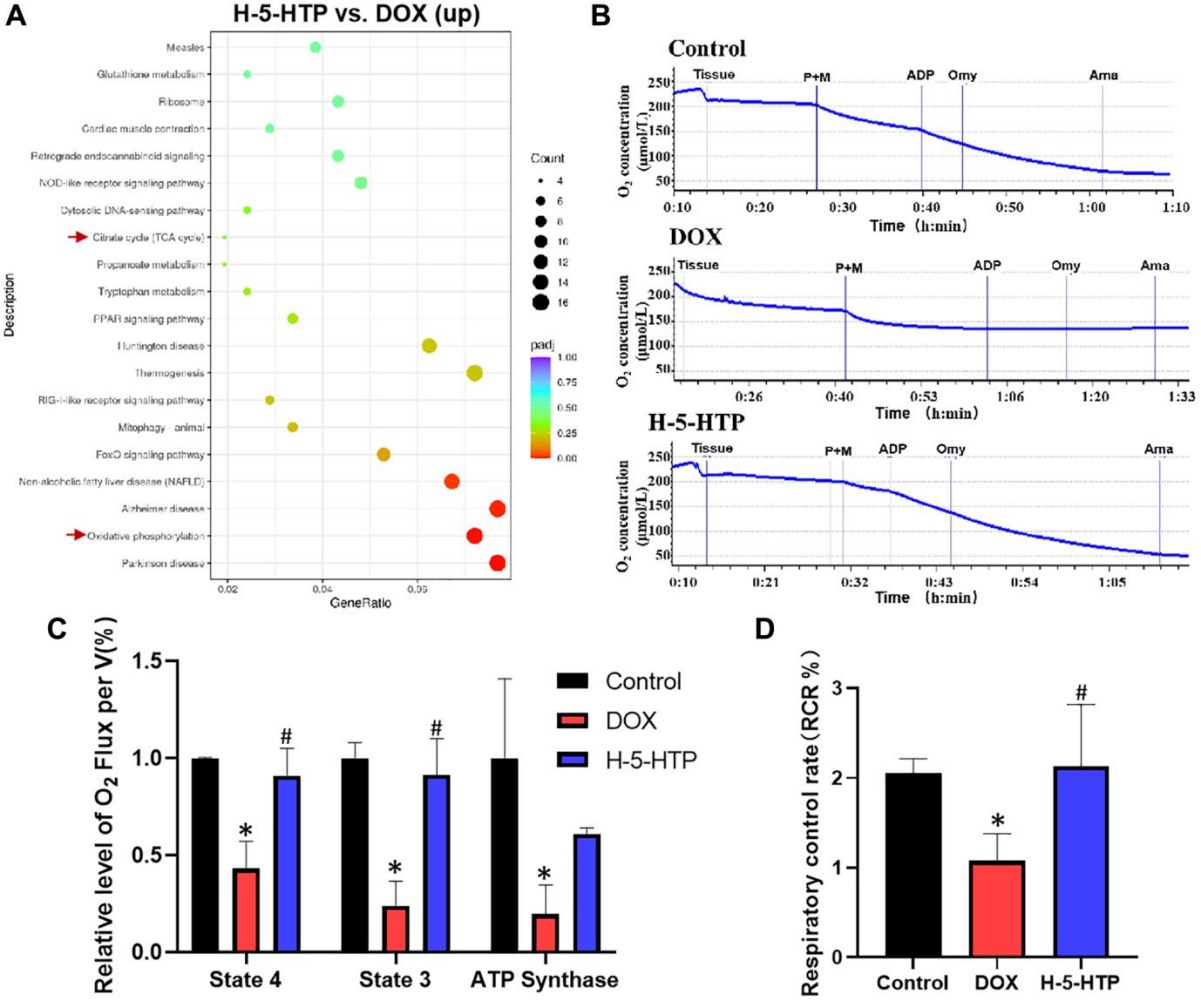
**5-HTP significantly improves doxorubicin-induced mitochondrial dysfunction in mice hearts.** (**A**) Scatter plot of KEGG enrichment analysis of upregulated genes by H-5-HTP group; (**B**) Changes in oxygen consumption in mouse heart tissue in the different intervention groups; (**C**) Respiration rate of mouse heart tissue in state 4, state 3 and after addition of oligomycin in different treatment groups; (**D**) Changes in control rate of mouse heart respiration. ^*^P < 0.05 vs. the Control group, ^#^*P* < 0.05 vs. the DOX group. (H-5-HTP group: H-5-HTP+DOX).

To elucidate the molecular underpinnings of 5-hydroxytryptophan (5-HTP) mediated cardioprotection against doxorubicin (DOX)-induced toxicity, we conducted a Kyoto Encyclopedia of Genes and Genomes (KEGG) enrichment analysis of genes that were upregulated in the H-5-HTP group relative to the DOX group (Figure 6A). This analysis revealed a significant upregulation of pathways related to oxidative phosphorylation and mitochondrial biogenesis in cardiomyocytes treated with 5-HTP, suggesting a potential mechanism by which 5-HTP confers its cardioprotective effects. Subsequently, we sought to determine whether the restoration of cardiac microenvironment stability by 5-HTP translates into improved cardiomyocyte function. Mitochondrial dysfunction is a well-documented contributor to the pathogenesis of DOX-induced cardiotoxicity [26], characterized by compromised energy production and elevated oxidative stress, culminating in apoptotic cell death [27]. To assess mitochondrial respiratory function, we employed the O2k mitochondrial respiration function assay system. As depicted in Figure 6B, heart tissues from the DOX-treated cohort exhibited a marked decrease in oxygen consumption, indicative of a suppressed mitochondrial respiration rate. Conversely, tissues from the 5-HTP-treated group demonstrated oxygen consumption patterns akin to those observed in the control group, suggesting a restoration of mitochondrial respiratory capacity by 5-HTP.

Further analysis of mitochondrial respiration in the presence of various substrates revealed that DOX treatment significantly impaired mitochondrial respiration in both state 3 (ADP-stimulated respiration) and state 4 (resting respiration), as well as diminished the respiratory control ratio (RCR), a measure of mitochondrial efficiency (Figure 6C, 6D). However, 5-HTP treatment appeared to bolster mitochondrial function within cardiac tissue, thus mitigating DOX-induced mitochondrial damage and re-establishing normal energy production. Moreover, the titration of oligomycin, an inhibitor of ATP synthase, provided insights into the impact of DOX on ATPase activity. The results indicated that DOX substantially reduced ATP synthase activity, thereby impairing the overall respiratory capacity of the mitochondria (Figure 6C). In stark contrast, 5-HTP treatment effectively ameliorated these effects, promoting the recovery of mitochondrial respiratory function and, by extension, cellular ATP production. Collectively, our findings underscore the potential of 5-HTP to counteract DOX-induced cardiotoxicity by enhancing mitochondrial function and preserving the cardiac microenvironment, thereby contributing to the maintenance of myocardial energy homeostasis (Supplementary Figure 1).

### 5-HTP does not interfere with the antitumor activity of DOX in MCF-7 and U2OS cells

**Figure 7:**
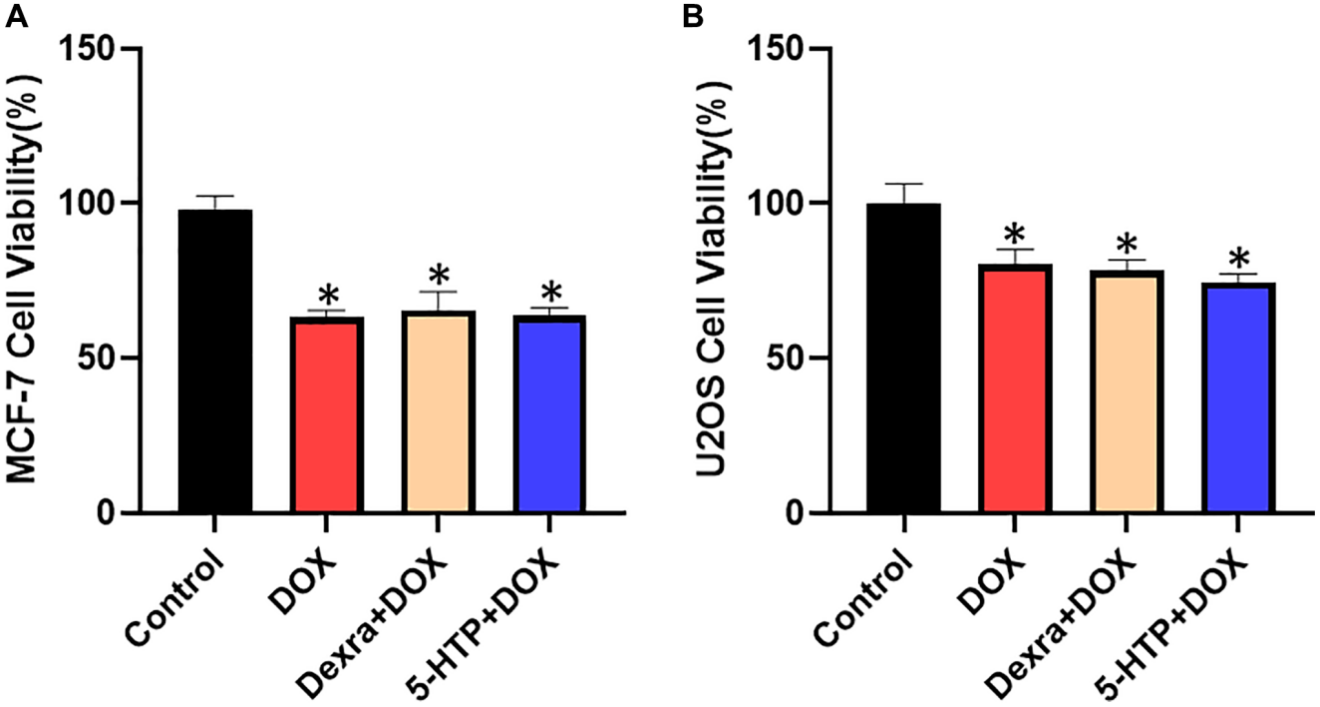
**Cell viability of MCF-7 and U2OS cells with different DOX and 5-HTP treatments.** (**A**) DOX-induced antineoplastic activities of 5-HTP in MCF-7 cells. (**B**) DOX-induced antineoplastic effects of 5-HTP in U2OS cells. ^*^P < 0.05 vs. the Control group, ^#^*P* < 0.05 vs. the DOX group.

To rigorously evaluate whether the co-administration of 5-hydroxytryptophan (5-HTP) compromises the antineoplastic efficacy of doxorubicin (DOX), we conducted a series of experiments to assess cell viability within MCF-7 (breast cancer) and U2OS (osteosarcoma) cell lines. Utilizing the Cell Counting Kit-8 (CCK-8) assay, a well-established method for the quantification of cell proliferation and cytotoxicity, we meticulously measured the survival rates of these cancer cells upon exposure to DOX, both with and without the adjuvant treatment of 5-HTP. Our comparative analysis revealed that 5-HTP, when administered independently, exhibited no discernible impact on the proliferation rates of both MCF-7 and U2OS cell populations, as evidenced by the similarity in survival rates to those of the control group. Crucially, the addition of 5-HTP to the DOX treatment regimen did not attenuate the cytotoxic properties of DOX; the cell survival rates in the combination therapy group paralleled those observed in the DOX-only group (refer to Figure 7A, 7B). These findings coalesce to suggest that 5-HTP does not possess inherent cytotoxic effects and, more pertinently, does not undermine the antitumor potency of DOX against MCF-7 breast cancer cells and U2OS osteosarcoma cells. In summary, our investigation supports the notion that 5-HTP serves as a non-interfering adjuvant, maintaining the integrity of DOX’s tumoricidal activity. This attribute nominates 5-HTP as a viable candidate for inclusion in chemotherapeutic regimens, potentially enhancing the therapeutic window of DOX by safeguarding against cardiotoxic side effects without compromising its cancer-targeting efficacy.

## DISCUSSION

A comprehensive elucidation of the mechanisms underlying the cumulative cardiotoxicity of anthracyclines, such as doxorubicin (DOX), is critical for devising strategies to mitigate the adverse cardiac effects associated with cancer chemotherapy. Our investigation delves into the progressive cardiac dysfunction induced by long-term DOX administration, drawing parallels with the pathophysiological processes observed in early myocardial infarction or ischemia-reperfusion injury. We observed that initial DOX exposure suppresses collagen synthesis, undermining the heart’s intrinsic repair mechanisms. This deficiency in the extracellular matrix (ECM) facilitates structural remodeling, characterized by ventricular wall thinning and dilatation, which precipitates a decline in cardiac function.

The cardiac milieu is critically dependent on gap junctions for the electrical coupling of cardiomyocytes, which facilitates the synchronized contraction of heart muscle [28, 29]. Beyond their role in electrical propagation, gap junctions are integral to autocrine and paracrine signaling cascades that underpin cellular metabolism, growth, and tissue equilibrium [12, 30, 31]. In the wake of focal cardiac injury, aberrations in electrical impulse generation and transmission are prevalent, with gap junctions serving as conduits for the dissemination of ischemic signals, contributing to extensive contractures and cellular demise [13, 32]. Accordingly, DOX-induced myocardial injury appears to perpetuate damage through these junctions by not only halting collagen synthesis but also by promoting the intercellular transmission of pathological signals such as calcium imbalance, heightened reactive oxygen species (ROS), and pro-inflammatory mediators, further undermining cardiac integrity.

Our findings reveal, for the first time, that 5-hydroxytryptophan (5-HTP), a biosynthetic precursor to the neurotransmitter serotonin, potently disrupts gap junction communication. The administration of 5-HTP curtailed the extension of chemotherapeutic drug-induced myocardial injury, attenuated cardiac inflammation, and oxidative stress, and notably ameliorated DOX-induced ventricular wall thinning, chamber enlargement, and the decline in ejection fraction, effectively restoring cardiac function. Employing the O2k mitochondrial function assay system, we quantitatively assessed cardiomyocyte metabolic competence, observing a significant augmentation in mitochondrial complex activity and ATP synthase function following 5-HTP treatment, indicative of a reversal in DOX-induced mitochondrial respiratory impairment. Crucially, 5-HTP did not detract from the antineoplastic efficacy of DOX.

When juxtaposed with Dexrazoxane (Dexra), an FDA-endorsed cardioprotective agent, 5-HTP not only emerged as a formidable gap junction inhibitor but also displayed a marginally superior efficacy in preserving cardiac structure. Dexra’s primary cardioprotective mechanism involves iron chelation, mitigating DOX-induced DNA damage [7, 9]. In contrast, our study implicates a distinct cardioprotective mechanism for 5-HTP. As an established pharmaceutical, 5-HTP’s safety profile is well-characterized, bolstering its potential as a cardioprotective adjunct in oncology treatment paradigms.

## CONCLUSIONS

In summary, our investigation has elucidated a novel cardioprotective role for 5-hydroxytryptophan (5-HTP) in the context of doxorubicin (DOX)-induced cardiac injury. We have demonstrated that 5-HTP confers a dual therapeutic advantage: it enhances mitochondrial bioenergetics and operates as an effective inhibitor of gap junction intercellular communication. By modulating these critical cellular pathways, 5-HTP effectively mitigates the expansion of myocardial injury that is precipitated by the degradation of the extracellular matrix (ECM) within the ventricular myocardium. Our findings suggest that 5-HTP holds considerable promise as an adjunctive treatment to safeguard cardiac function during the administration of DOX, offering a potential strategy to alleviate the cardiotoxic side effects associated with this potent chemotherapeutic agent.

## Supplementary Materials

Supplementary Figure 1
