# Supplementary material for: 5-Hydroxytryptophan acts as a gap junction inhibitor to limit the spread of chemotherapy-induced cardiomyocyte injury and mitochondrial dysfunction
Source: Aging (Albany NY). 2024 Mar 10;16(5):4889–903. doi: 10.18632/aging.205641 (PMC10968683; doi:10.18632/aging.205641)
Supplement: Supplementary Figure 1 [file aging-16-205641-s001.pdf]

SUPPLEMENTARY FIGURE

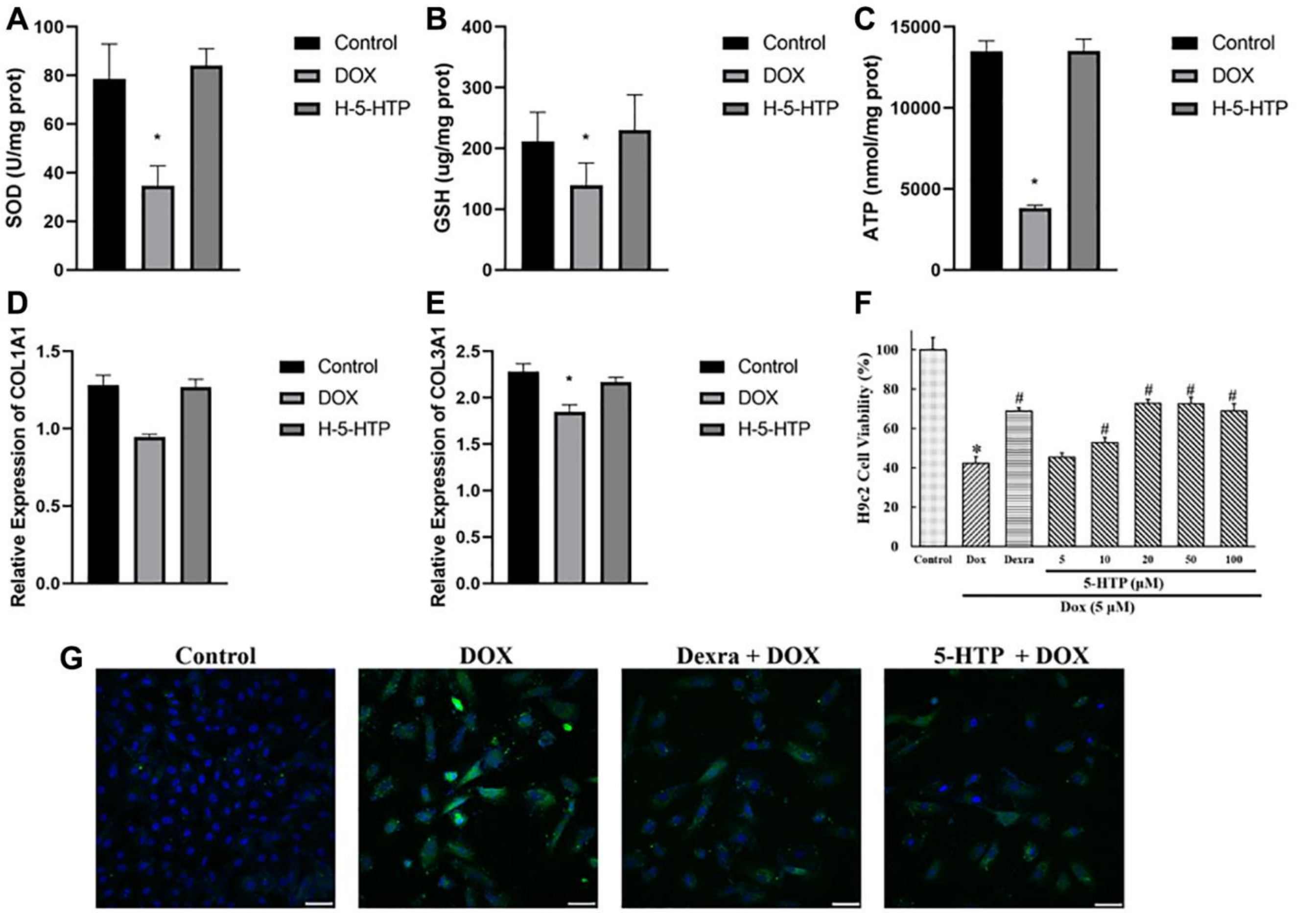

Supplementary Figure 1. Effects of different treatments on myocardial cell biochemistry and gene expression. (**A**–**C**) Depict the concentrations of Superoxide Dismutase (SOD), Glutathione (GSH), and Adenosine Triphosphate (ATP), respectively, in myocardial cells following various treatments. (**D**, **E**) Depict the relative RNA expression levels of collagen type I alpha 1 chain (COL1A1) and collagen type III alpha 1 chain (COL3A1) after different treatments. (**F**) Illustrates the cell viability of myocardial cells as assessed by CCK-8 assay, including statistical significance of differences observed. (**G**) Presents immunofluorescence staining for ROS in myocardial cells subjected to different treatments, using a specific probe.
